# Risk Factors and Prevention of Viral Hepatitis-Related Hepatocellular Carcinoma

**DOI:** 10.3389/fonc.2021.686962

**Published:** 2021-09-09

**Authors:** Xinhe Zhang, Lin Guan, Haoyu Tian, Zilu Zeng, Jiayu Chen, Die Huang, Ji Sun, Jiaqi Guo, Huipeng Cui, Yiling Li

**Affiliations:** ^1^Gastroenterology Department, The First Affiliated Hospital of China Medical University, Shenyang, China; ^2^The 3rd Clinical Department of China Medical University, Shenyang, China

**Keywords:** hepatocellular carcinoma, hepatitis B virus, hepatitis C virus, risk factors, prevention

## Abstract

Hepatocellular carcinoma (HCC) is a common cancer in the world, and its incidence is increasing yearly. Hepatitis B virus (HBV) infection and hepatitis C virus (HCV) infection are important causes of HCC. Liver cirrhosis, age, sex, smoking and drinking, and metabolic risk factors will increase the risk of cancer in HBV/HCV patients. And viral load, APRI, FIB-4, and liver stiffness can all predict the risk of HCC in patients with viral infection. In addition, effective prevention strategies are essential in reducing the risk of HCC. The prevention of HCC involves mainly tertiary prevention strategies, while the primary prevention is based on standardized vaccine injections to prevent the occurrence of HBV/HCV. Eliminating the route of transmission and vaccination will lead to a decrease in the incidence of HCC. Secondary prevention involves effective antiviral treatment of HBV/HCV to prevent the disease from progressing to HCC, and tertiary prevention is actively treating HCC to prevent its recurrence.

## Introduction

Liver cancer is the main cause of cancer-related deaths worldwide. According to the latest report of the International Agency for Research on Cancer of the World Health Organization in 2020, the global incidence of liver cancer is 910,000, accounting for 4.7% of all cancers, and rank sixth in incidence among cancers, while the number of deaths is ranked third worldwide (1). Hepatocellular carcinoma (HCC) is the most common type of liver cancer. In 2017, there were 10.1 cases per 100,000 people worldwide, with the highest incidence in Asia and Africa (2). The incidence rate in Africa is 24.2 cases per 100,000 individuals, and the incidence rate in East Asia is 35.5 cases per 100,000 individuals. In the 2015 Asia-Pacific region, the number of deaths from HCC accounted for 72.7% of deaths from liver disease (3). China accounts for more than 50% of the world’s cancer burden, and the mortality rate of the HCC population is second only to that of lung cancer (4). The etiology of HCC is currently unclear, and may be the result of a synergistic influence of multiple factors. HCC etiology is mostly believed to be associated with liver cirrhosis, viral hepatitis, alcoholic liver disease, metabolic-related fatty liver disease, and aflatoxin infection. Among these, viral hepatitis is the most important factor, of which chronic hepatitis B (HBV) and chronic hepatitis C (HCV) infections are the most common. Approximately 80% of HCC cases worldwide are caused by HBV or HCV (5). In 2018, approximately 360,000 cases of HCC were caused by chronic HBV infection, accounting for 55% of the total number of HCC cases, while chronic HCV infection caused approximately 160,000 cases of cancer, mainly from HCC (6). In addition, the annual incidence of HCC in patients with liver cirrhosis caused by chronic HBV or HCV infection is 2%–5% (6). The reason for the increasing incidence of HCC in patients with hepatitis can be attributed to the fact that very few people undergo routine hepatitis testing, thus most patients are unaware of that they have been infected. Only a small number of patients with hepatitis are regularly screened for liver cancer. Due to the hidden onset of HCC, more than 80% of patients with liver cancer are diagnosed in the middle and advanced stages. Especially for patients with HBV/HCV coinfection, compared with HCV infection alone, liver cirrhosis and HCC are more likely to occur (7).

Compared with HBV infection, HCV infection increases the risk of HCC by 15-20 times. Once the patient progresses to the stage of cirrhosis, the annual incidence of HCC is 2%-4% (8). HBV is a DNA virus (9), and its genome consists of a double-stranded circular DNA with a length of approximately 3200 nucleotides. HBV DNA escapes the body’s immunity through genetic mutations and uses DNA polymerase-reverse transcriptase to replicate in immune cells to influence immune cell functions, which stimulate the T cell-mediated immune response of the host to combat infection. However, because the body does not clear HBV completely, HBV persists in a stable form and HBV DNA can be integrated into host DNA. Some gene products (HBx, pre-S/S protein) can activate proto-oncogenes and re-activate HBV virus replication, inhibit tumor suppressor genes, resist apoptosis, induce neovascularization, decrease the stability of the host genome, induce liver cell regeneration, promote the process of liver cirrhosis, and thus, play an important role in the occurrence of HCC (10). The progression of HBV into HCC is an intertwined and complex process, in which the protein X (HBx protein) encoded by HBV plays an important role. HBx protein can indirectly activate the C-FOS proto-oncogene through the JAK/STAT signaling pathway (11), and it can also inhibit the activity of the p53 promoter to down-regulate the mRNA levels of p53, and forms a complex with the P53 protein in the cytoplasm to inhibit its metastasis to the nucleus. These proto-oncogenes and tumor suppressor genes play an important role in the occurrence of HCC (12). HBx protein can regulate non-coding RNA to enhance the migration and invasion of HCC cells. HBx can also induce abnormal lipid metabolism by activating the P2X-Akt pathway, and alter the normal glucose metabolism process of the liver by activating the transcription factor Nrf2 (13). The integration of HBV DNA into the host genome is another important mechanism. HBV can integrate its genome into the host chromosome, causing host chromosome instability, leading to changes in gene expression and protein translation. The inserted HBV sequence often contains an X gene with a carboxy-terminal deletion, which can cause the expression of a truncated 17K-Da HBx protein leading to the formation of HCC (14). TERT plays an important role in promoting tumorigenesis. It is also the most frequently integrated gene with HBV, which by combining with the core promoter region of TERT, exerts a regulatory effect on the transcriptional activity of the TERT promoter (15). Random integration of HBV DNA can lead to chromosome structure deletion, amplification, or inversion, resulting in the instability of the host genome. The clonal integration of AAV2 was identified in 11 of 193 cases of HCC, with a total frequency of 5%. A small region of AAV2, including the 3’-inverted tandem repeat region, when integrated in proximity of cancer genes such as telomerase reverse transcriptase (TERT), cyclin A2 (CCNA2), cyclin E1 (CCNE1), and tumor necrosis factors (ligands) superfamily, can induce gene expression and promote cell proliferation and malignant transformation (16).

HCV is an RNA virus, and its genome is a single-stranded positive-stranded RNA with a length of approximately 9500 nucleotides (17). Because HCV RNA can easily mutate, it can continue to replicate in the body by escaping the body’s immune attack by mutation. Among the viral proteins, the core protein can induce the expression of iNOS and COX-2 to up-regulate the level of ROS in hepatocytes, inducing the occurrence of oxidative stress (18). At the same time, HCV RNA can promote the expression of COX-2 at the transcriptional level by oxidative stress and NF-KB pathway. Oxidative stress is an important mechanism for the occurrence of HCC. The expression of iNOS and COX-2 are closely related to vascular epidermal growth factor (VEGF). Through the COX-2/PGE2/EP/VEGF pathway, PGE2 can recruit myeloid derived suppressed cells to impaired Tregs and CD8+ T cell function, which can promote the proliferation of liver cancer cells and tumor blood vessels (19). In addition, the dysregulation of signaling pathways occurs in the development and progression of HCC, including cell proliferation and differentiation, and angiogenesis. The proteins encoded by HCV are directly involved in the pathways related to Wnt/β-catenin, Hedgehog, and tyrosine kinase receptors (20). Overexpression of COX-2 can promote the expression of YAP mRNA and protein by activating the Wnt/β-catenin signaling pathway. In turn, YAP can promote the transcription of COX-2 and promote the proliferation of liver cancer cells through the positive feedback loop (21). COX-2/PGE2 induces the expression of MMP-2 and MMP-9 and down-regulates the expression of E-cadherin by activating the β-catenin signaling pathway, thereby enhancing the invasion and migration ability of liver cancer cells (22). However, due to insufficient replication, it cannot stimulate the body’s immune clearance. Thus, it remains in the body for an extended period. HCV and HBV cause HCC *via* different mechanisms and gene products may indirectly affect cell proliferation and differentiation to induce HCC.

## Risk Factors

### Liver Cirrhosis

Liver cirrhosis is the main risk factor for HCC in patients with hepatitis. In some developed countries, the mortality rate of HCC-related cirrhosis is increasing (23). **Table 1** lists the risk factors for liver cirrhosis caused by hepatitis in various contexts, of which liver cirrhosis is the most frequent (24–33). Compared with patients with HBV, patients with HCV seem to be more likely to develop liver cirrhosis. According to a US census, the proportion of HCV infections among liver cirrhosis and HCC cases continued to rise between 2001 and 2013. The incidence of liver cirrhosis ranged from 159 to 193 per 100,000 patients, and the mortality rate varied from 83 cases to 126 cases per 100,000 patients, mainly caused by HCV. The incidence of liver cancer increased from 17 cases to 45 cases per 100,000 patients, an increase of 2.5 times (34).

**Table 1:** Risk factors of different guide.

Factor	Guide	Year	Risk factors
HBV	KASL (24)	2019	age, liver cirrhosis
	JSH (25)	2019	age, cirrhosis, high viral load, hcc family history, PLT
	AASLD (26)	2018	age, cirrhosis, HCC family history
	EASL (27)	2017	cirrhosis, inflammation, age, sex, alcohol, hepatitis, HIV, diabetes, metabolic syndrome, smoking, HCC family history, high viral load, HBV genotype
	APASL (28)	2015	age, sex, alcohol, hepatitis, cirrhosis, HCC family history, high viral load, aflatoxin
HCV	TASL (29)	2020	cirrhosis, HCC family history, sex, age, alcohol, diabetes, low albumin, low platelet, AST, ALT, GGT
	AASLD (30)	2019	alcohol, cirrhosis, leukemia
	APASL (31)	2016	HCV genotype, cirrhosis, obesity
	JSH (32)	2016	age, PLT, ALT, cirrhosis
	CASL (33)	2015	cirrhosis

KASL, The Korean Association for the Study of the Liver; JSH, Japan Society of Hepatology; AASLD,Association for the Study of Liver Diseases; EASL, European Association for the Study of the Liver; APASL, Asian-Pacific Association for the Study of the Liver; TASL, the Taiwan Liver Cancer Association and the Gastroenterological Society of Taiwan; CASL, the Canadian Association for the Study of the Liver; PLT, platelet; HCC, hepatocellular carcinoma; ALT, alanine aminotransferase; AST, aspartate aminotransferase; GGT, gama-glutamyl transpeptadase.

### Age and Sex

HCC rarely occurs before the age of 40, and the peak period is approximately 60 years old. Males have an increased incidence of HCC after hepatitis virus infection, which is 2 to 3 times higher than that of females (35). Adiponectin can activate AMP-activated protein kinase and p38 to prevent the development of liver cancer, although male testosterone activates the JNK protein in adipocytes to inhibit the secretion of adiponectin (36). In females, estrogen may reduce viral transcription and the inflammatory response, control the progression of viral infections, and plays a protective role in the occurrence of HCC. It can also affect the occurrence of liver cancer by regulating mRNA levels, DNA methylation, and histone modifications in liver tissue (37).

### Race

In addition to sex differences, the differences among populations of different races are also of note. A survey in the United States explored the correlation between the race of patients infected with HCV infection and the risk of future HCC. The results indicated that the annual incidence of HCC varied from high to low. For Hispanics, non-Hispanic whites, and African Americans (7.8%, 4.7%, and 3.9%, respectively) (38), racial differences are likely attributable to differences in risk factors between ethnic groups and geographic locations. Currently, there are few studies on ethnic differences in different regions, and extensive epidemiological investigations are needed.

### Blood Type

Chronic hepatitis B patients with different ABO blood types have different risks of developing HCC. According to research by Chen et al., the risk of HCC increases by 39% in women with blood type A, while women with blood types AB and B have a lower risk. Hepatitis B patients with blood type A are more likely to exhibit liver damage and liver cirrhosis (39). ABO blood type has been reported to be associated with a variety of cancers, including gastric cancer and pancreatic cancer. However, the relationship between different ABO blood types and HCC is still being further explored. Geographic differences indicate that more people with non-O blood types will have a higher prevalence of HCC and even non-O blood types are poor prognostic indicators for HCC patients after hepatectomy (40–42). For HCC related to viral hepatitis, the risk of different ABO blood types is different. According to the study by Chen et al., patients with blood type A have an increased risk of HCC by 39%, while women with blood type AB and B have a lower risk of developing the disease. In a survey in China, it was also found that compared with O-blood subjects, the adjusted odds ratio for HCC in A-blood HCV patients was 3.301. This difference may be related to HCC cytokines. Changes in the levels of these cytokines have an important impact on the occurrence and development of tumors. Furthermore, in different ABO blood types, the binding of affinity of EGF and EGFR is different, with the expression of A antigen significantly altering the binding properties of the protein and affecting cell signal transduction and growth (39). In addition, the concentration of serum sICAM-1 in patients with blood type A is significantly reduced. The combination of sICAM-1 with circulating cells can inhibit the combination of lymphocytes and endothelial cells. The reduction of its concentration can enhance the basic resistance of cancer cells to apoptosis and promote tumors (41). However, the relationship between ABO blood type and HCC is uncertain. Some studies believe that there is no correlation between ABO blood type and HCC risk, and some studies believe that blood type B increases the risk of HCC. This may be related to the difference in etiology of HCC in different studies (43, 44). Thus, further research and exploration are needed.

### Smoking and Drinking

Smoking and drinking are the main lifestyle risk factors for HCC, especially when combined with concomitant viral infections. The combined effect will accelerate the occurrence of HCC. Smoking may increase liver toxicity and aggravate hepatitis in patients by increasing the hepatitis B virus load and augmenting the toxic substances resulting from smoking (45, 46). Liver damage accelerates the occurrence of HCC. Tobacco contains numerous carcinogens, including cadmium. Its relationship with HCC has also been clearly reported (47). Epidemiological studies suggest that smoking is a minor risk factor for the development of HCC, and the adjusted relative risk of liver cancer for smokers is 1.51 (8). In addition, a study with a small sample population showed that postoperative smokers had significantly worse RFS rates than non-smokers (*P*=0.024). The RFS of heavy smokers is significantly lower than that of non-smokers and light smokers (*P*<0.001) (48), but this conclusion requires investigation in a larger sample population, and there is still controversy about whether smoking is a risk factor for HCC.

Long-term drinking stimulates increases in NF-kB activity, which is an inflammation-related tumor factor, which promotes the expression of cell adhesion molecules and other tumor-promoting and metastatic molecules. A survey showed that there is little difference between mild to moderate drinking and the clinical outcome of patients with chronic HBV infection. Heavy drinking will significantly accelerate the development of liver cancer, and the risk increases from 1.3 to 8.4 times (49). Alcohol consumption plays an extremely important role in HCC caused by HCV infection. A British study conducted a multifactor analysis of 1620 patients with chronic HCV infection and found that chronic HCV infection caused liver cirrhosis mostly due to alcoholism. The attribution score reached 36%, while for patients with a history of alcohol abuse, this score exceeded 50% (50). The higher the alcohol intake, the greater is the risk of HCC.

### Metabolic Risk Factors

Metabolic risk factors, such as obesity, diabetes, high triglycerides, and high blood pressure, increase the risk of HCC in patients with hepatitis infection. A large population survey showed that the incidence of metabolic syndrome in the HCC population was significantly higher than that in the control group (37.1% and 17.1%) (51). A large cohort study in Taiwan explored the relationship between HBV and HCC in detail. The researchers included data on 1690 Taiwanese male public officials aged 40–65 years with HBV infection and characterized by different metabolic risk factors. There were significant differences in the incidence of HCC involvement in carriers. The incidence of HCC in patients with more than three metabolic risk factors (13.60%) was significantly higher than that in patients with few metabolic risk (4.83%) (52). The specific mechanisms involved are currently not clear. It is generally recognized that fatty acids are mainly catabolized in the liver. Hepatocytes convert triglycerides formed by fatty acid esterification into low-density lipoproteins and release them. Obese and hyperlipidemic patients have higher amounts of fatty acids entering the liver, which exceeds the metabolic capacity of the liver, leading to the deposition and accumulation of a large number of fat droplets (53). The production of reactive oxygen species increases, and mitochondrial oxidative stress is induced. These processes can lead to inflammation in the liver, necrosis and regeneration, hepatic stellate cell activation and liver fibrosis to promote liver cancer (54). Free fatty acids can also increase insulin resistance and directly promote hepatocellular carcinogenesis by regulating cell growth and migration (55). In patients with metabolic risk factors, the level of leptin in the body is often higher than that in normal individuals (56). An increase in the leptin content can easily promote the release of tumor necrosis factor-, which can interact with nuclear factors to promote liver cell apoptosis and proliferation. Tumor necrosis factor- is an important proinflammatory factor leading to HCC.

## Risk Prediction

### HBV

The incidence of HCC increases with increasing serum HBV DNA levels. A prospective cohort study included 3653 participants (aged 30–65 years old) who were seropositive for hepatitis B surface antigen. A total of 164 cases of liver cancer occurred and 346 cases reported deaths. The incidence of HCC showed a dose-response relationship with the increase in serum HBV DNA levels at the beginning of the study. With HBV DNA levels from less than 300 copies/mL to 1 million copies/mL or higher, the incidence of HCC ranged from 108 to 1152 per 100,000 people-years. The corresponding cumulative incidences were 1.3% and 14.9%, respectively (57). For the prediction threshold of HBV DNA, in the REVEAL study, when the serum HBV DNA levels were 2000 IU/mL, the risk of HCC was significantly increased. For patients with liver cirrhosis, the risk of HBV DNA levels not reaching 2000 IU/mL is still very high (58). However, current studies indicate that low-level viremia can also cause HCC (59). Low-level viremia (LLV) is defined as a level of HBV DNA continuously or intermittently greater than the detection limit but less than 2000 IU/mL. A Chinese study observed 78 patients treated with entecavir therapy, and low levels of serum HBV DNA were associated with the progression of fibrosis, which is a risk factor for HCC. Compared with patients with reversal of liver fibrosis, patients with progression of fibrosis had a lower virological response rate during follow-up. The viral load was between 20 and 200 IU/mL (60). In another retrospective analysis of 875 patients with chronic HBV infection who received entecavir monotherapy, the probability of HCC in LLV patients during treatment was significantly higher than that in patients with continuous response, and the hazard ratio was 1.98, which is even more pronounced in patients with liver cirrhosis (61). Therefore, it is important to continue to pay attention to LLV during treatment (62, 63).

The lower the baseline hepatitis B surface antigen (HBsAg) value, the lower is the risk of HCC. Tseng et al. found that HBsAg can be used to predict the risk of HCC in HBe-negative patients with a low viral load. The researchers investigated 1688 patients with chronic HBV infection. The univariate analysis showed that the risk ratio of HCC in patients with more than 100 IU/mL was 5.4 compared with the others. The result of multivariate analysis was that a high HBsAg level was an independent risk factor for HCC (HR: 13.7) (64). For the HBsAg prediction cutoff value, the most recent meta-analysis including 12,541 patients among 10 studies showed that the OR value of a high HBsAg level and low HCC level was 4.99 below the cutoff value of 100 IU/mL, but the variance was high. Under the threshold of 1000 IU/mL, the combined OR of a high HBsAg and low HCC level was 2.46, but the variance was low. Therefore, an HBsAg level of more than 100 IU/mL, especially more than 1000 IU/mL, is associated with an increased risk of liver cancer (65).

Serum markers can also effectively predict the risk of HCC. The gradient of alanine aminotransferase (ALT) levels (low-normal-temporary abnormal-persistent abnormality) was significantly related to the risk of HCC (P < 0.001) (66). The AST and platelet index (APRI) and fibrosis-4 index (FIB-4) are commonly used to predict the risk of HCC in patients with hepatitis infection. The APRI has high specificity and low sensitivity, but FIB-4 has low specificity and high sensitivity. Thus the combined predictive effect of both is superior (58).

HBV carriers with FIB-4 exceeding 1.29 will have a significantly increased risk of HCC (67). However, there are also studies showing that FIB-4 has higher diagnostic performance for liver fibrosis in patients with chronic hepatitis virus infection than the APRI (68). The combination of the APRI and FIB-4 may stratify the HCC risk of LLV patients with chronic HBV infection. When both the APRI (0.5) and FIB-4 (1.45) scores were high, one high and one low, or both low, the accumulated incidence of HCC for 5 years was reported to be 13.9%, 1.4% and 1.2%, respectively (58). However, the conclusion of this study still needs to be further explored. In addition, detection of microRNA molecules in the blood may also be a way to predict the risk of HCC (69).

### HCV

Noninvasive markers can predict the risk of HCC in patients with HCV (70). APRI and FIB-4 are commonly used indicators. FIB-4 over 2.18 can predict the occurrence of HCC in patients with HCV with a sensitivity of 92.4% and specificity of 87.2%. Compared with the APRI, FIB-4 has a higher diagnostic performance for predicting HCC caused by HCV infection (71).

Shear wave elastography (FibroScan) is a common technique to measure the degree of liver fibrosis. Its application has developed rapidly given its advantages of convenience, noninvasiveness, and economical cost. It has been reported that there is a correlation between liver stiffness (LSM) and the risk of HCC (72). Shear wave elastography (FibroScan) is a common technique to measure the degree of liver fibrosis. Its application has developed rapidly given its advantages of convenience, noninvasiveness, and economical cost. It has been reported that there is a correlation between liver stiffness (LSM) and the risk of HCC (73). However, the LSM value alone cannot accurately predict the risk of HCC in patients with HCV infection. A case matching study showed that the combination of LSM, LSM interquartile range, ALT, and AFP showed good diagnostic performance in both the experimental group and the verification group (AUROCs were 0.86 and 0.8), which allowed the prediction of the incidence liver cancer noninvasively and with high accuracy.

### Risk Prediction Models

Risk prediction models are often used to predict the risk of HCC in patients with HBV. **Table 2** lists the risk prediction models of HCC in HBV (74–81). George V. Papatheodoridis summarized the risk scoring models developed by scholars over the past 10 years. These scoring models divide the risk of HCC in patients with HBV into three levels: low, medium and high. Among them, low-risk patients do not need regular HCC monitoring, while medium-high risk patients require regular monitoring. Ten prediction models predicted the risk of HCC in untreated patients with HBV, 90% of which included age as a variable. Researchers use the AUROC to judge the accuracy of the model. REACH-B, LSM-HCC, and D2AS can predict low-risk groups more accurately. Seven predictive models predict the risk of HCC in patients with HBV treated with nucleotide analogs with high accuracy (82).

**Table 2:** Risk prediction model.

	REACH-B	mREACH-B	GAG-HCC	CU-HCC	LSM-HCC	RWS-HCC	PAGE-B	APA-B
sex	*	*	*			*	*	
age	*	*	*	*	*	*	*	*
ALT	*	*						
ALB				*	*			
TBIL				*				
PLT				*			*	*
AFP						*		*
HBeAg	*	*						
HBV DNA	*		*	*	*			
Liver stiffness		*			*			
cirrhosis			*	*		*		
AUC for predicting 5-year risk	0.699	0.806	0.88	0.76	0.83	–	–	0.862

*Represents related content of risk models.

## Prevention

**Figure 1:**
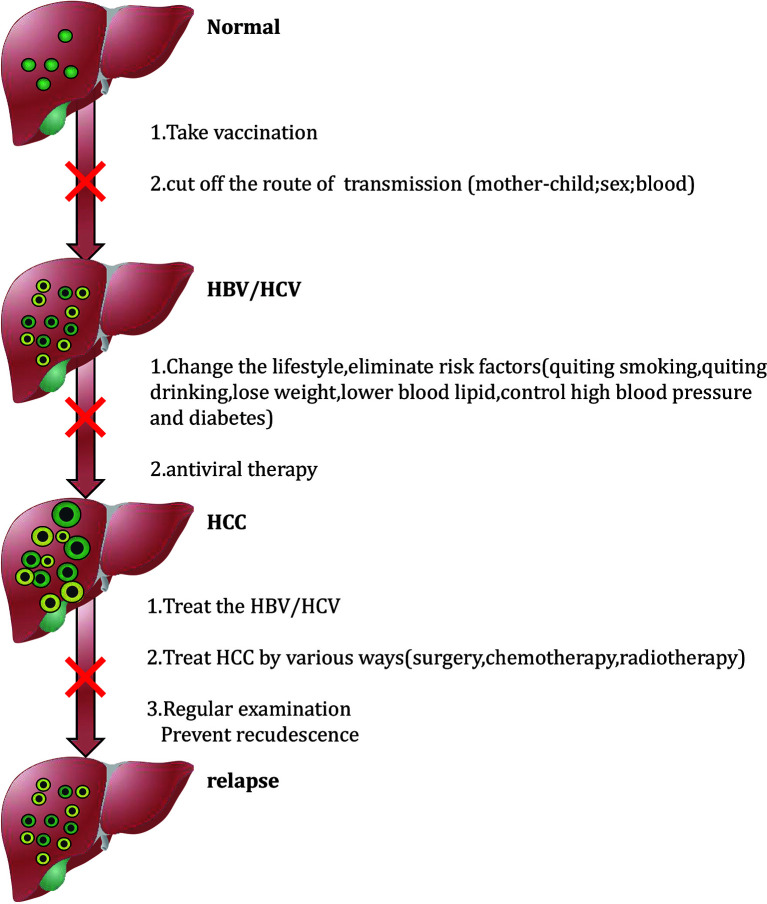
Flow diagram of prevention.

Since the diagnosis of HCC is often at an advanced stage, treatment options are limited at this time, and the prognosis is poor, it is urgent to formulate effective preventive measures to improve the prognosis of patients by preventing the occurrence of HCC. HCC prevention measures are divided into primary, secondary and tertiary levels. Primary prevention refers to the use of behavior modification and activities that are conducive to maintaining overall good health to prevent risk factors associated with liver disease to avoid the risk of HCC. Secondary prevention refers to changing personal behavior and reducing the body’s exposure to high-risk for liver disease. Through screening and monitoring, tertiary prevention can accurately identify and diagnose existing infections among patients with liver disease to promote early detection and timely intervention of HCC or minimize the risk of HCC recurrence (83). **Figure 1** briefly summarizes tertiary prevention strategies, and the following content will be described in detail.

### Prevention of Viral Infections

Prevention of viral infections can include blocking the transmission route and vaccination. Hepatitis B vaccination is the most important approach to prevent HBV infection. The hepatitis B vaccine is a purified hepatitis B surface antigen. After vaccination, it stimulates the immune system to produce protective antibodies. Preventing chronic HBV infection through vaccination can reduce the risk of HCC by 85% (84). Many adults ignore the need for hepatitis B vaccination. People who are negative for hepatitis B surface antibodies and antigens should receive the hepatitis B vaccine in a timely manner. The United Sates Food and Drug Administration approved the Heplisav-B vaccine to prevent HBV infection in adults. The difference between Heplisav and the currently approved hepatitis B vaccine is that its adjuvant is a Toll-like receptor 9 agonist that can induce hepatitis B surface antigen-specific antibody effects against HBV infection (85). However, for patients coinfected with HBV and HCV, the response to HBV vaccination is usually weak. The specific mechanism is unclear. It is possible that CD4(+) T cells overexpress KLRG1, which interferes with the response of CD4(+) T cells towards new antigens through the p16ink4a and p27kip1 pathways and induces an immune senescence state (86). Second, greater attention should be paid to hindering the transmission of hepatitis B. Mother-to-child transmission during pregnancy is the main mode of hepatitis B transmission (87, 88). Regarding the treatment plan of HBV during pregnancy in different countries, tenofovir is the first choice for antiviral treatment of mothers from early to late pregnancy. The European Association for Liver Research recommends (27) for those who have received nucleoside analogue (NA) treatment. Pregnant women should continue to use TDF, and entecavir or other NA should be changed to TDF. For all pregnant women with high HBV DNA levels (20,000 IU/mL) or HBsAg levels (4log10 IU/mL), prevention of TDF should start from 24–28 weeks of pregnancy and continue until 12 weeks after delivery. In the United States (89), patients in the immune tolerance period or in the inactive (low replication) state of chronic hepatitis B infection can be monitored without treatment until the third trimester. For patients who do not breastfeed, antiviral therapy can be stopped in the fourth week after delivery. If breastfeeding is planned, antiviral treatment can be stopped at delivery. For pregnant women with HBV DNA content higher than 6–7 log 10 IU/mL, the Asia-Pacific region recommends tenofovir treatment from 28 to 32 weeks of pregnancy (28). Mothers with HBV during pregnancy should not be restricted from breastfeeding. Although breast milk contains HBV, the risk of HBV infection in breastfed infants is very low, not higher than that of infants fed with formula milk. But the premise is that the baby is vaccinated at birth. The WHO recommends that no matter what the mother’s hepatitis B virus status is, the vaccinated baby should be breastfed (90), but if the mother is in the NA treatment period, breastfeeding is not encouraged.

The main preventative approach to HCV infection is to prevent the route of transmission. The transmission route of HCV is usually horizontal transmission, including those who receive a contaminated blood transfusion or use blood products, inject drugs, receive solid organ transplants without HCV screening, share toothbrushes with HBV-infected patients, have a partner suffering from AIDS (91). The research campaign of hepatitis C vaccines is under continuous study. However, because HCV can easily mutate, no clinical universally preventable vaccine has been found at this time. Most vaccines currently under evaluation are used to treat HCV infection. Peptide vaccines can present vaccine peptides to T cell receptors through human leukocyte antigen (HLA) molecules, thereby inducing HCV-specific T cell responses. In 2009, a Japanese study described vaccine derived from a peptide from the HCV core protein. Cellular and humoral responses of HCV-positive patients with different HL)-I-A alleles were found. Through a phase I clinical trial including 22 HCV-positive patients, after 12 injections of vaccines, specific IgG in the plasma of 15 patients increased. Due to its tolerance and higher immune enhancement rate, this program is recommended for phase II studies to study its clinical efficacy (92). A phase II clinical trial in 2015 explored the safety and efficacy of personalized peptide vaccines (PPVs) in patients with HCV-positive advanced HCC. PPVs were used to treat patients with HCV-positive advanced HCC. Only 3 out of 42 patients had peptide-specific CTL responses before vaccination, but peptide-specific CTL responses were observed in 23 out of 36 patients after vaccination. The peptide-specific IgG response was also enhanced in 19 out of 36 patients (93). In 2018, the Pasteur Institute in Iran found that the Hp91 peptide is a promising vaccine candidate for HCV therapy through *in vivo* mouse models. The response of HCV nonstructural protein 3 (NS3) plays an important role in clearing acute HCV infection. Hp91 peptides can enhance the specific humoral and cellular immunity of mouse NS3 (94). In addition to peptide vaccines, DNA vaccines, cell culture vaccines, and viral vector vaccines have been proposed (95). However, the development of hepatitis C vaccine still requires additional clinical trials and greater exploration.

Sharing needles for intravenous injection is the main risk factor for serious HBV and HCV infections. Sharing of unsterilized injection equipment can lead to the spread of HCV and HBV among injecting drug users, but it can be prevented to a large extent through the Needle Exchange Program (NEP) (96, 97). The NEP is a disease prevention project aimed at preventing blood-borne diseases as the main purpose of the injection of intravenous drug users by sharing syringes (98). NEP started in the Netherlands in 1984. To prevent the hepatitis B epidemic among intravenous drug users, it has been adopted by many countries. A 4-year prospective cohort study was conducted in a population of 2,860 intravenous drug users. The results of the study found that the needle exchange program could significantly reduce the risk of participating in injections (99). The services provided by NEP include HBV vaccination. Alanko used a 10-year survey to vaccinate drug-injecting populations for hepatitis B vaccine: 74.8% of the population received basic vaccines. The vaccination plan was responsive and after up to three booster doses, 84.5% of the population reached the level of protective anti-HBs, proving the feasibility of HBV vaccination in the service provided by NEP (100).

### Lifestyle Changes

Lifestyle plays a key role in HCC, including weight control and the maintenance of a healthy diet, especially comprising fruits and vegetables, which can reduce the incidence of HCC. It is necessary to avoid drinking and smoking, to control hypertension and diabetes through medication, and to maintain normal circulating blood sugar and cholesterol levels (101).

### Antiviral Treatment

Antiviral therapy can not only reduce the occurrence of HCC but also prevents the recurrence of HCC. Anti-hepatitis drug therapy is the most basic treatment for HBV/HCV infection, and it also plays an important role in preventing the occurrence of HCC. Anti-HBV drugs include entecavir, adefovir, tenofovir, lamivudine, telmivudine, and other nucleotide analogs (NAs). The 2013 Asia-Pacific Association for the Study of Liver Diseases (APASL) reported that nucleotide drug therapy can significantly reduce HBV DNA levels and reduce the risk of HCC. The application of anti-hepatitis drugs to patients with HCC can also significantly improve survival. At present, many studies have compared 5 types of NAs to reduce the risk of HCC. A meta-analysis in 2013 showed that all NAs could significantly reduce the incidence of HCC, with no significant difference in the reduction ability of the different drugs (102). The latest 2019 AASLD meeting announced 4 studies exploring the efficacy of entecavir and tenofovir in reducing the risk of HCC. The studies were from Taiwan, the United States, Canada, and included a meta-analysis (103). All the studies indicated that tenofovir treatment was more effective than entecavir. If liver cirrhosis develops or does not respond to NA treatment, it is more likely to progress to HCC during NA treatment in patients with HBV infection (104). In addition, as tertiary prevention, NAs can reduce the risk of hepatitis B-related HCC recurrence (105).However, A study explored the genes of 73 HCC patients with previous HBV but no HCV infection, and 81 HCC patients with chronic HBV. The results were found in 11 previous HBV-free HCV cases and 61 (75.3%). Clonal HBV integration events have also been identified in chronic HBV cases (106). Therefore, from a genetic standpoint, despite the serum clearance of hepatitis B surface antigen, there is still a risk of HCC. The currently preferred drugs for hepatitis C are direct antiviral drugs (DAAs). According to several reports, DAA treatment and response can reduce the occurrence of HCC (107, 108). There is some controversy regarding studies that have found that statins can reduce HCC caused by HCV infection by 49%, and whether these agents can be used clinically to reduce the risk of HCC is still being explored (109). DDAs mainly target NS3/4A protease, NS5B polymerase, and the NS5A replication complex during HCV replication. At present, DAA drugs for the treatment of hepatitis C are divided into pan-genotypes and gene-specific types. Pan-genotypes include sofosbuvir/vipatavir, gcarevir/pirentavir, and klopavir. In phase III clinical trials, sofosbuvir/vipatavir was administered for 12 weeks, the SVR rate in patients with 6 HCV genotypes was between 97% and 100% (110). The SVR rate of 6 genotypes were greater than 99% with the gcarevir/pirentavir combination after 12 weeks (111). In addition, there are different DAA drugs for different genotypes. According to the consensus of the 2016 Asia-Pacific Conference, for HCV GT-1 patients, these patients should receive ledipasvir/sofosbuvir for 12 weeks. Patients with HCV GT-2 and HCV GT-3 genotypes are recommended to use Sofosbuvir combined with ribavirin. the ledipavir/sofosbuvir combination can be used for patients with genotypes 4, 5, and 6. In addition, no IFN treatment should be used for patients with decompensated liver disease (112).

Interferon drugs (PEG-IFN), as immunomodulators, not only have antiviral effects but also have certain antitumor and antiangiogenic effects. A retrospective study in Taiwan, China, found that PEG-IFN was better at reducing the incidence of HCC caused by HBV infection. The cumulative incidence of HCC in the 5-year treatment of HBV infection with PEG-IFN was significantly lower than that of the NA group (113), although another study did not find any difference between PEG-IFN and DAA in reducing the risk of HCC caused by HCV (114). The risks of HCC development after HCV eradication are similar between patients treated with peg-interferon plus ribavirin and direct-acting antiviral therapy). For patients with HCC, adjuvant IFN after radical treatment of hepatitis-related liver cancer can improve the survival rate. IFN can also reduce the recurrence rate of patients with HCC with tumors less than 3 cm in diameter (115).

After the hepatitis C virus is cleared, some patients are still at risk of developing HCC. This is mainly due to the decrease in reactive immune surveillance caused by the rapid decline in viral load (116). An Italian research group analyzed patients with liver cirrhosis who received DAA treatment, and the incidence of HCC was 3.1% in 6 months after treatment (117). Later, different countries used retrospective or prospective studies to explore the incidence of HCC in different time periods after DAA treatment. The most recent review summarized the exploration of the occurrence of HCC after antiviral therapy, and summarized the factors (i.e., male, liver cirrhosis, diabetes, AFP levels, high liver stiffness, and past history of HCC) that contribute to the higher incidence of HCC (118). Among them, the cut-off value of liver stiffness value is still under further exploration. Rinaldi et al. used Fibroscan to measure the basic liver stiffness. The risk of HCC in patients exceeding 30 kPa was significantly increased (*P*=0.019). The cutoff value was determined by ROC curve analysis to be 27.8 kPa (119). Pons et al. used regression analysis to divide patients into low-risk groups and high-risk groups. The low-risk group was defined as the liver elasticity hardness measurement value of <10 kPa or 10-20 kPa and serum albumin ≥4.4 g/dL at the 1-year follow-up of treatment. The risk population was the measured value of liver elasticity ≥20 kPa or 10-20 kPa and serum albumin <4.4 g/dL at the 1-year treatment follow-up (120, 121). This is a new tool for estimating the risk of HCC after HCV is cured, and it needs to be further explored in the clinic.

In addition to conventional antiviral drugs, there are currently some drugs that can prevent the occurrence of HCC, of which aspirin, silybin, and metformin are the most frequently studied. Aspirin is the most common antiplatelet drug and can reduce the risk of HCC in patients with HBV. A cohort study included 204,507 patients with HBV in Taiwan from 1997 to 2012. Continuous follow-up surveys found that the incidence of HCC in patients who took aspirin daily for 5 years was lower than that of patients who did not use aspirin. After adjusting for other related risk factors, aspirin treatment was associated with a 29% reduction in the risk of HCC (122). The mechanism by which aspirin prevents HCC is complex and has not been fully explored. The recognized mechanism is to inhibit the proinflammatory cyclooxygenase 2 (COX-2) enzyme, which is involved in the development of liver cancer. COX-2 oxidizes arachidonic acid, stimulates the production of inflammatory factors, promotes cell growth and angiogenesis, inhibits tumor apoptosis, and enhances tumor cell invasion. In addition, aspirin can increase the expression of Bax in HepG2 cells to reduce Bcl-2 protein expression and promote the apoptosis of liver cancer cells (123).

Silybin has long been used as a hepatoprotective drug that has membrane stability and antioxidant activity. It directly inhibits liver fibrosis and the activation of various cytokines in hepatic stellate cells. It can also significantly reduce tumor cell proliferation, angiogenesis and insulin resistance (124). It can even quickly and safely reduce HCV viremia (125).

In addition, animal experiments have shown that mice taking metformin exhibit a reduction in HCC of approximately 57%. It may prevent the occurrence of HCC, which may inhibit the occurrence of cancer by activating the LKB1/AMPK pathway and inhibiting the expression of the fatty acid synthases ACC and FASN (126).

According to the chronic hepatitis B guidelines issued by the World Health Organization in 2015 (127), it is recommended that high-risk groups should undergo AFP and ultrasound examinations every 6 months to monitor the occurrence of HCC, including patients with liver cirrhosis, patients with family history of HCC, and HBV DNA over 40 years old among non-cirrhotic patients with levels exceeding 2000 IU/mL. For patients who do not need antiviral therapy, it is recommended to monitor serum ALT, HBeAg, and HBV-DNA levels at least once a year to continuously assess the stability of the disease. According to the 2017 Asia-Pacific clinical practice guidelines, separate AFP monitoring is not recommended. For non-cirrhotic HBV patients, the age of the high-risk population provides a more detailed classification, with women over 50 years old in Asia, over 40 years old in men, and over 20 years old in Africa having a higher risk of HCC (128). For HCV patients, even after reaching SVR, HCV-infected patients without cirrhosis are still at risk of HCC, fibrosis stage (F2 or 3), advanced age, γ-glutamyltransferase (γGT) level, and DM is non-hepatic. Patients with cirrhosis have a high risk of HCC, and these patients should receive closer attention for the development of HCC after SVR.

### Early Diagnosis of HCC

Early diagnosis of HCC is very important. Serum AFP detection is a commonly used and important method for early diagnosis of HCC, but its low sensitivity and specificity limit the diagnostic efficiency. Therefore, there is an urgent need for new biomarkers (129). The current common abnormal coagulation proenzyme (DCP) and alpha-fetoprotein heterogene 3 (AFP-L3), although the sensitivity of single detection of the three indicators is not high, the three simultaneous detections can significantly improve the diagnostic efficiency. Currently, Japan has included the three indicators in a HCC staging system, which has been used for the stratification and prognosis of liver cancer patients (130). New biomarkers include golgi protein 73 (GP73), glypican 3 (GPC-3), osteopontin (OPN), and insulin growth factor II (IGF-II) (131, 132). In addition, detection of circulating in serum miRNA can monitor the occurrence of liver cancer, including miR-193a-3p, miR-369-5p, miR-672, miR-429 and let-7i, with the advantages of being non-invasive, stable, and simple. However, their application is still premature and has not yet entered the clinic on a large scale (133). Circulating tumor cells (CTC) are tumor cells that shed and metastasize from primary solid tumors to the circulatory system. They play a vital role in the process of tumor metastasis. The number of CTCs in the peripheral blood of patients with HCC is significantly greater than that in patients with non-malignant liver diseases. CTC are more effective than AFP in diagnosing HCC, and combined detection can increase sensitivity (AUC=0.821) (134). At present, growing evidence indicates that it can be used as an effective indicator for HCC diagnosis, staging, prognosis prediction, and recurrence detection (135). When cancer cells rupture and die, they release contents such as circulating tumor DNA (ctDNA), and these ctDNA genome fragments circulate in the blood. The analysis of ctDNA opens up new possibilities for solid tumor assessment through liquid biopsy specimens, but the biological characteristics of many aspects of these ctDNA are still not very clear.

### HCC Treatment

Treatment of HCC mainly depends on the patient’s state and disease progression. Patients with early HCC can choose treatment methods such as liver resection, transplantation, and radiofrequency ablation, while patients with locally advanced stages can undergo interventional treatments such as transcatheter arterial chemoembolization (TACE). For patients with advanced HCC, the prognosis is poor and there is no cure. The research and development targets of new drugs for HCC therapy are related to signaling pathways and molecular mechanisms prevalent in HCC. Important advances in HCC biology have revealed there are several changes in a variety of molecular signaling pathways. The receptor tyrosine kinase pathway activates multiple growth factor receptors (VEGF, EGFR, and IGF receptor) that activate downstream signaling pathways including the PI3K/AKT/mTOR pathway, the Ras/Raf/MEK pathway, and the Wnt/β-Catenin signaling pathway, which promote tumor cell proliferation and angiogenesis (136). The most researched and developed therapeutic targets involve the VEGFR, which is also the pathway that has entered the most clinical trials. VEGF is a key angiogenic factor that plays an important regulatory role in HCC. Sorafenib is the earliest targeted drug, and its therapeutic targets include VEGFR, PDGFR, and Ras/Raf/MEK/ER (137). Its activity involves the inhibition of a variety of kinase inhibitors that inhibit the cell signaling pathways of target site and tumor blood vessels. With the continuous progress of research, small molecule type V tyrosine kinase inhibitors lenvatinib and atilizumab combined with bevacizumab have achieved better results than sorafenib and because of their superior advantages, have quickly become the standard first-line treatment. According to the latest ESMO clinical practice guidelines, for patients with portal vein invasion and extrahepatic metastasis, atilizumab combined with bevacizumab is the first-line treatment option (138). In terms of combined use, the remission rate of lenvatinib combined with pembrolizumab in the phase Ib trial reached 46%, although further exploration is needed (139). Nonetheless, about 20% of patients still lack suitable responses; thus, further second-line treatment is needed, including combination with regorafenib, cabozantinib, ramucirumab, pembrolizumab, and nivoliuzumab. Apatinib is a new generation small molecule anti-tumor angiogenesis agent. It has a highly selective and potent inhibitory effects on VEGFR2 receptors. Recent phase III clinical trials have shown that the risk of disease progression or death following apatinib treatment is reduced by 52.9% Apatinib is currently approved for patients with advanced HCC who have failed or become intolerable after receiving at least one prior first-line systemic treatment (140). In addition, there have been different discussions on agents targeting other molecular pathways. Sirolimus or everolimus has now entered phase III clinical trials (141). These agents mainly inhibit the mTOR pathway to prevent tumor progression. It has entered phase III clinical trials. Phase clinical trials have found that sirolimus will not improve the long-term recurrence-free survival (RFS) rate of liver transplantation in HCC patients for more than 5 years, but the advantages are significant in the first 3–5 years (142). DEAH-box polypeptide 32, PFK118-310, PFK115-584, and CGP049090 all act on the Wnt/β-Catenin pathway to treat HCC, but there is no clinical trial available to date (143, 144). Combination therapy plays an important role in the treatment of HCC. A multi-center study showed that adjuvant (131I)-metuximab after radical resection of liver cancer could significantly increase the RFS rate and improve the prognosis of patients (145). However, whether drug treatment is needed before or after surgery is still under dispute.

MicroRNAs (miRNA) are a type of small non-coding RNA, which can promote tumor development and inhibit tumor progression by combining with target gene mRNA to act on coding genes. High expression of miRNA has been associated with the promotion of tumor cell proliferation and differentiation. MiR-93 and miR-106b contribute to the tumorigenesis of HBV-related HCC (146). MiR-106b activates epithelial-mesenchymal transition (EMT), up-regulates the expression of RhoGTPases to promote tumor cell migration, and down-regulates APC Activation of the Wnt/β-catenin pathway enhances the proliferation of HCC. PDCD4 is a target gene of miR-93 and its expression is down-regulated by miR-93, which promotes HCC proliferation and EMT-mediated invasion and metastasis (147). In addition, miR-1470 promotes HCC cell proliferation and inhibits apoptosis of tumor cells by down-regulating the ALX4 gene (148). Conversely, the low expression of tumor suppressor miRNA also plays an important role in HCC. MiR-21 is one of the first miRNAs found to be associated with tumors. It inhibits the invasion and metastasis of HCC through its target gene PTEN. The Let-7 family affects key regulator of the cycle by inhibiting oncogenes to exert tumor suppressor genes and anti-tumor proliferation effects. MiR-125b-5p reduces HCC proliferation by inhibiting sirtuin 7 and inhibits HCC malignancies by targeting SIRT6 (149). MiR-1297 can target EZH2 to inhibit the proliferation of liver cancer cells and promote the apoptosis of liver cancer cells (150). MiR-627-5p exerts an effect by inhibiting BCL3 and miR-122 can significantly down-regulate Bcl-W protein to promote liver cancer cell apoptosis (151). By transfecting miR-122 adenovirus to the mouse liver cells, the tumor mass was significantly reduced (152). MiRNA as a target for HCC treatment are also under further exploration. MiRNAs that promote or supplement a tumor suppressor miRNA that is abnormally low expressed in tumor tissue, or inhibits the cancerous target gene of the tumor miRNA are particularly attractive approaches. MRX34 was the first cancer target miRNA drug, a liposome-based miR-34 mimic, and was proposed to supplement the expression of miR-34 in HCC and inhibit tumor progression (153). It was stopped for use in phase I clinical trials because of severe immune-mediated adverse reactions, although it presented a rationale for HCC treatment (154). Another approach is to inhibit the abnormally high expression of cancerous miRNA in tumors. By applying oligonucleotide technology to silence highly expressed miRNAs in tumor tissues, it is possible to inhibit tumor cell growth and or metastasis. Antisense oligonucleotides can selectively bind to and block miR-221 activity during transplantation of human liver cancer into mice. Treatment with miR-221 can significantly reduce its levels in tumors and normal liver samples, causing the activity of tumor suppressor genes (P27, P57 and PTEN) to increase by 3 times (155). In addition, treatment with miRNA can influence the activity of chemotherapeutic drugs. miR-193b mimics can promote an increase in sensitivity to sorafenib and promote its efficacy in HBV-related HCC patients (156). Certain drugs can also regulate miRNA levels. For example, after arsenic trioxide acts on liver cancer cells, the expression of miRNA-29 increases, up-regulating the expression of the tumor suppressor gene p53, and inhibiting liver cancer cells (157).

## Discussion

At present, HCC is still a cancer with high prevalence and mortality. Prevention and treatment of viral hepatitis and minimizing risk factors exposure are crucial elements in decreasing the global burden of HCC. A large number of risk prediction models and risk factors have been developed to predict the risk of HCC in patients with viral infection. However, most of them are from Asia and the threshold value is still under further study. And a large sample is required for further verification in the possibility of HCC after anti-hepatitis. Standardized vaccine injections can decrease the incidence rate of illness, but there is no effective vaccine for hepatitis C virus for primary prevention. In addition, although new preventive drugs are still being explored, further elucidation and confirmation are still needed.

## Publisher’s Note

All claims expressed in this article are solely those of the authors and do not necessarily represent those of their affiliated organizations, or those of the publisher, the editors and the reviewers. Any product that may be evaluated in this article, or claim that may be made by its manufacturer, is not guaranteed or endorsed by the publisher.

## Author Contributions

Conceptualization, LG and YL. Methodology, XZ and JC. Validation, ZZ, HT, and HC. Curation, JG, JS, and DH. Writing – Original Draft Preparation, XZ. Writing – Review & Editing, YL. Visualization, YL. Supervision, YL. Project Administration, YL. Funding Acquisition, YL. All authors contributed to the article and approved the submitted version.

## Conflict of Interest

The authors declare that the research was conducted in the absence of any commercial or financial relationships that could be construed as a potential conflict of interest.

## Publisher’s Note

All claims expressed in this article are solely those of the authors and do not necessarily represent those of their affiliated organizations, or those of the publisher, the editors and the reviewers. Any product that may be evaluated in this article, or claim that may be made by its manufacturer, is not guaranteed or endorsed by the publisher.
